# Characterisation of AMB-FUBINACA metabolism and CB_1_-mediated activity of its acid metabolite

**DOI:** 10.1007/s11419-022-00649-3

**Published:** 2022-10-28

**Authors:** Hunter D. J. Webb, David B. Finlay, Shuli Chen, Andrea J. Vernall, Eric Sparkes, Samuel D. Banister, Rhonda J. Rosengren, Michelle Glass

**Affiliations:** 1 Department of Pharmacology and Toxicology, University of Otago, Dunedin, 9016 New Zealand; 2 Department of Chemistry, University of Otago, Dunedin, 9016 New Zealand; 3 Lambert Initiative for Cannabinoid Therapeutics, Brain and Mind Centre, The University of Sydney, Sydney, NSW 2050 Australia; 4 School of Chemistry, The University of Sydney, Sydney, NSW 2006 Australia

**Keywords:** AMB-FUBINACA, Synthetic cannabinoid receptor agonist, Human liver microsomes, Carboxylesterase 1, Carboxylic acid metabolite, Cannabinoid receptor

## Abstract

**Purpose:**

AMB-FUBINACA is a synthetic cannabinoid receptor agonist (SCRA) which is primarily metabolised by hepatic enzymes producing AMB-FUBINACA carboxylic acid. The metabolising enzymes associated with this biotransformation remain unknown. This study aimed to determine if AMB-FUBINACA metabolism could be reduced in the presence of carboxylesterase (CES) inhibitors and recreational drugs commonly consumed with it. The affinity and activity of the AMB-FUBINACA acid metabolite at the cannabinoid type-1 receptor (CB_1_) was investigated to determine the activity of the metabolite.

**Methods:**

The effect of CES1 and CES2 inhibitors, and delta-9-tetrahydrocannabinol (Δ^9^-THC) on AMB-FUBINACA metabolism were determined using both human liver microsomes (HLM) and recombinant carboxylesterases. Radioligand binding and cAMP assays comparing AMB-FUBINACA and AMB-FUBINACA acid were carried out in HEK293 cells expressing human CB_1_.

**Results:**

AMB-FUBINACA was rapidly metabolised by HLM in the presence and absence of NADPH. Additionally, CES1 and CES2 inhibitors both significantly reduced AMB-FUBINACA metabolism. Furthermore, digitonin (100 µM) significantly inhibited CES1-mediated metabolism of AMB-FUBINACA by ~ 56%, while the effects elicited by Δ^9^-THC were not statistically significant. AMB-FUBINACA acid produced only 26% radioligand displacement consistent with low affinity binding. In cAMP assays, the potency of AMB-FUBINACA was ~ 3000-fold greater at CB_1_ as compared to the acid metabolite.

**Conclusions:**

CES1A1 was identified as the main hepatic enzyme responsible for the metabolism of AMB-FUBINACA to its less potent carboxylic acid metabolite. This biotransformation was significantly inhibited by digitonin. Since other xenobiotics may also inhibit similar SCRA metabolic pathways, understanding these interactions may elucidate why some users experience high levels of harm following SCRA use.

## Introduction

**Fig. 1:**
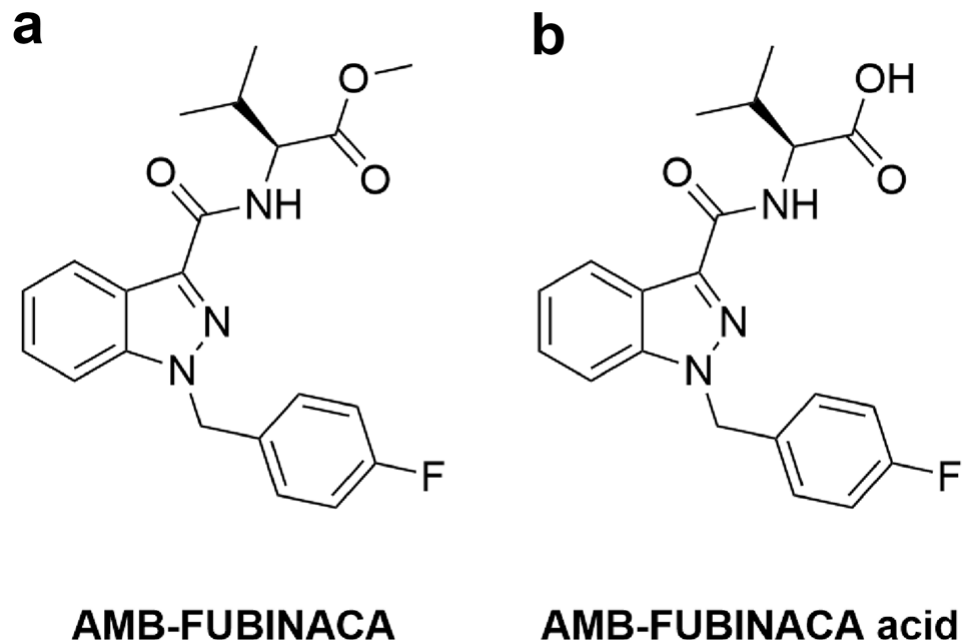
Chemical structures of **a** AMB-FUBINACA and **b** AMB-FUBINACA carboxylic acid metabolite

As of September 2021, 1079 new psychoactive substances were reported by the United Nations Office on Drugs and Crime, approximately 29% of which were classified as synthetic cannabinoid receptor agonists (SCRAs). Methyl (2*S*)-2-((1-((4-fluorophenyl)methyl)indazole-3-carbonyl)amino)-3-methylbutanoate (AMB-FUBINACA; Fig. 1a) is an SCRA that was associated with at least 64 deaths in Auckland, New Zealand, between 2017 and 2019 [1]. While little is known about its mechanism of toxicity, its psychoactive effects are mediated through the cannabinoid receptor type-1 (CB_1_)_._ AMB-FUBINACA exhibits high binding affinity (p*K*_i_ 8.72 ± 0.12) for CB_1_, and high potency and efficacy in multiple downstream signalling pathways, including inhibition of cAMP signalling, pERK activation, receptor internalisation and β-arrestin-1 and -2 translocation [2]. In contrast to SCRAs, the primary psychoactive constituent of *Cannabis sativa*, delta-9-tetrahydrocannabinol (Δ^9^-THC), acts as a partial agonist at CB_1_, with approximately 80-fold lower potency than AMB-FUBINACA in common assay systems [2, 3]. In humans, Δ^9^-THC-mediated partial agonism of CB_1_ results in euphoria, cognitive impairments, feelings of dizziness and visual changes [4], and typically results in few if adverse effects. SCRAs, however, are more toxic, and can produce effects such as psychosis, respiratory depression, cardiac arrest, seizures, suicidal ideation and, in extreme circumstances, death [1, 5–7]. Non-CB_1_-mediated effects have been demonstrated, including cellular toxicity and necrosis; toxic metabolite generation causing mitochondrial damage and loss of cellular membrane integrity; and thermal degradant production, such as cyanide generation following the combustion of carboxamide SCRAs [8–10]. Therefore, toxicity induced by AMB-FUBINACA may be a result of its high affinity and efficacy towards CB_1_, but could also occur through other independent molecular targets. Furthermore, while metabolites of many SCRAs retain partial agonist activities at CB_1_ [11–13], activity of the AMB-FUBINACA carboxylic acid metabolite (Fig. 1b), the primary metabolite of AMB-FUBINACA [14], has not previously been reported.

CYP3A4 is the most abundant cytochrome P450 (CYP450) isoform in the liver [15] and is involved in the metabolism of approximately 70% of all therapeutic drugs [16]. Some SCRAs such as AB-CHMINACA [17], AKB-48 [18], EAM-2201 [19] and STS-135 [20] are primarily metabolised by CYP3A4. Thus, inhibition of CYP3A4 may increase both the CB_1_-mediated and non-CB_1_-mediated activities of these compounds. This can easily occur following exposure to other drugs such as furanocoumarins, itraconazole, ketoconazole, clarithromycin, erythromycin, nefazodone, and ritonavir, as well as through grapefruit juice consumption [21–23]. This highlights the importance of understanding the metabolism of SCRAs for prediction of drug–drug interactions. Interestingly, of the fatal AMB-FUBINACA cases in New Zealand, *p*-fluorophenylpiperazine (pFPP), Δ^9^-THC, ethanol (alcohol), psychiatric medications, and cotinine (a nicotine metabolite derived from tobacco) were present in 43, 41, 42, 48 and 88% of the cases, respectively [1]. Thus, it is possible that the toxic events elicited by AMB-FUBINACA in New Zealand were related to a pharmacokinetic or pharmacodynamic interaction between AMB-FUBINACA and these other compounds.

Pharmacokinetic interactions could be linked to the fact that hepatic metabolism of AMB-FUBINACA is extremely rapid, primarily producing AMB-FUBINACA (carboxylic) acid, as this demethylated metabolite is the most important in the biotransformation pathway [24]. Not only is this biotransformation rapid, but also AMB-FUBINACA acid represents more than 99% of all metabolites produced in experiments using human liver microsomes (HLM) [14]. To date, the metabolic enzyme(s) responsible for the rapid hepatic conversion of AMB-FUBINACA to AMB-FUBINACA acid remain unknown, although carboxylesterase 1 (CES1) was one of several candidates proposed by Presley et al. [25]. AMB-FUBINACA is largely stable in human plasma with ~ 86% of the original concentration remaining intact following a five-hour in vitro incubation. Notably, 94% remained in the presence of esterase inhibitors [26]. The absence of CESs in human blood may explain why in vitro plasma studies demonstrate AMB-FUBINACA to be very stable, despite the metabolism being classified as rapid [20, 26, 27]. No AMB-FUBINACA was detected in the whole blood or urine of non-fatal poisonings with AMB-FUBINACA [28], and only 26% of AMB-FUBINACA fatalities in New Zealand had AMB-FUBINACA present in their blood [1]. This may suggest that plasma protein binding displacement interactions, alongside systemic circulation resulting in transport of AMB-FUBINACA to the liver, play an important role in metabolism of this SCRA. Therefore, the current study aimed to identify the main metabolising enzymes associated with AMB-FUBINACA metabolism. Experiments were performed in the presence and absence of the CYP450 cofactor NADPH to determine the involvement of CYP450 enzymes in the biotransformation of AMB-FUBINACA to AMB-FUBINACA acid. Recombinant enzymes were also utilised to specifically identify the primary metabolising enzyme as well as its ability to be inhibited with known inhibitors. Lastly, the activity of AMB-FUBINACA acid at CB_1_ was compared to the parent compound as an indicator of how metabolism could potentially impact CB_1_-mediated effects in humans.

## Materials and methods

### Drugs, standards, and reagents

AMB-FUBINACA, methyl (2*S*)-2-((1-((4-fluorophenyl)methyl)indazole-3-carbonyl)amino)-3-methylbutanoate (C_21_H_22_FN_3_O_3_, MW: 383.423 g/mol) and AMB-FUBINACA acid, methyl (2*S*)-2-((1-((4-fluorophenyl)methyl]indazole-3-carbonyl)amino)-3-methylbutanoic acid (C_20_H_20_FN_3_O_3_, MW: 369.396 g/mol) were synthesised and analytically characterised at the Lambert Initiative for Cannabinoid Therapeutics, Brain and Mind Centre, and The School of Chemistry, The University of Sydney, and both were stored at 10 mM in dimethyl sulfoxide (DMSO) at -80 °C. Digitonin (C_56_H_92_O_29_, M: 1229.3 g/mol) and telmisartan (C_33_H_30_N_4_O_2_, M: 514.6 g/mol) were purchased from Cayman Chemical (Ann Arbor, MI, USA) and stored at 10 mM in DMSO and 1 mM in high performance liquid chromatography (HPLC) grade methanol, respectively. Δ^9^-Tetrahydrocannabinol, (6a*R*,10a*R*)-6,6,9-trimethyl-3-propyl-6a,7,8,10a-tetrahydro-6*H*-benzo[*c*]chromen-1-ol was purchased from THC Pharm GmbH (Frankfurt, Germany) and stored at 31.6 mM in absolute ethanol at -0 °C.

HPLC grade methanol (CH_4_O, M: 34.04 g/mol) and acetonitrile (C_2_H_3_N, MW: 41.05 g/mol) were purchased from VWR (Radnor, PA, USA). Potassium dihydrogen orthophosphate (KH_2_PO_4_, MW: 136.09 g/mol) was purchased from ThermoFisher Scientific (Waltham, MA, USA). Bovine serum albumin (BSA) was purchased from Life Technologies New Zealand Limited (Auckland, NZ) and prepared in 0.9% (w/v) saline, daily and kept on ice. β-NADPH, nicotinamide adenine dinucleotide phosphate tetrasodium salt (C_21_H_26_N_7_Na_4_O_17_P_3_, MW: 833.4 g/mol) was purchased from Roche (Basel, Switzerland) and prepared in Milli-Q^®^ water, daily, and kept on ice. Sodium chloride (NaCl, MW: 58.44 g/mol) was purchased from Labchem International (Heidelberg, Germany). Trifluoroacetic acid (C_2_HF_3_O_2_, MW: 114.02 g/mol) was purchased from Sigma-Aldrich (St. Louis, MO, USA). HLM pooled from 50 donors were purchased from ThermoFisher Scientific. CES1 isoform b (CES1b/CES1A1) human recombinant, expressed and purified from *Bacillus thuringiensis israelensis* (BTI) insect cells, carboxylesterase-2 (CES2) human recombinant, expressed in baculovirus infected BTI insect cells and *p*-nitrophenyl acetate (PNPA; C_8_H_7_NO_4_, MW: 181.15 g/mol) were purchased from Sigma-Aldrich.

For radioligand binding [^3^H]CP55,490 was purchased from PerkinElmer (Waltham, MA, USA), V‐well plates from Hangzhou Gene Era Biotech Co. Ltd. (Zhejiang, China), and harvest plates (GF/C filters, 1.2 µm pores) from PerkinElmer. The vacuum manifold was purchased from Pall Corporation (Port Washington, NY, USA). Ultima Gold XR scintillation fluid was purchased from PerkinElmer and radioactivity was detected in a MicroBeta2^®^ TriLux Liquid Scintillation Counter and was also purchased from PerkinElmer.

For cAMP inhibition assays, cultureware was purchased from Corning (Corning, NY, USA), 96-well CulturPlates were purchased from PerkinElmer, coelenterazine-H was purchased from Nanolight Technologies (Pinetop, AZ, USA), V-well polypropylene dispensing plates were purchased from Innovative Laboratory Products (Phoenix, AZ, USA), and forskolin was purchased from Cayman Chemical. All other cell culture reagents and media were purchased from ThermoFisher Scientific.

### Instrumental analysis

All chromatographic runs were carried out using a Prominence high performance liquid chromatography (HPLC) system (Shimadzu, Kyoto, Japan), comprising a CBM-20A HPLC modular system controller, a DGU-20A3R degassing unit, dual LC-20AR solvent delivery modules, a SIL-20ACHT autosampler and a CTO-20AC column oven. An SPD-M20A UV/VIS photodiode array detector (DAD; Shimadzu) was used for detection. Full spectra were recorded in the range of 190–800 nm. Chromatographic separations were achieved using a Phenomenex^®^ (Torrance, CA, USA) Gemini^®^ (5 μm, C18, 110 Å, 4.6 × 150 mm) 714,145–14 column. Equipment control, data acquisition and peak integration were performed with LabSolutions software (Shimadzu). A 26-min binary gradient was developed with 0.05% (v/v) trifluoroacetic acid in water (A) and 0.05% (v/v) trifluoroacetic acid in 9:1 acetonitrile/water (B). The binary gradient was held at 10% B for 0–0.5 min, increased to 60% B from 0.5 to 2.5 min, increased to 80% B from 2.5 to 15.0 min, decreased to 10% B from 15.0 to 17.0 min before a final isocratic hold from 17.0 to 26.0 min, with the flow rate set at 1 mL/min. The Phenomenex^®^ C18 column was maintained at 40 °C and the injection volume was 20 μL. The ultraviolet detection of AMB-FUBINACA and AMB-FUBINACA acid was performed at 299 nm.

## In vitro metabolism

### CYP450 microsomal incubations

The in vitro metabolism protocol was derived from Apirakkan et al. [29] and Menzies et al. [30] and optimised to include good practice of in vitro metabolism experimental recommendations [31]. BSA was used to reduce non-specific binding of AMB-FUBINACA to labware and concentrations were derived from glucuronidation assays in Allan et al. [32]. Phosphate buffer (100 mM; pH 7.4) and BSA (0.2% w/v) were combined, and NADPH (1 mM) was added to microsomal experiments requiring CYP450 activation; the tubes were kept on ice at all times. HLM (0.25 mg/mL) were added, and the solution was mixed and preincubated for 2 min at 37 °C. Reactions were initiated by addition of AMB-FUBINACA (3 μM) and the total reaction volume was 300 μL. AMB-FUBINACA metabolism was quenched at 20, 40, 60 and 120 s by the addition of 150 μL of ice-cold methanol. Either digitonin (150 μM) or telmisartan (50 μM) was included in subsequent experiments in the absence of NADPH to determine if AMB-FUBINACA metabolism could be inhibited. Concentrations of digitonin and telmisartan were selected based on previously established inhibition of CES1 and CES2 in HLM (data not shown). Digitonin or telmisartan were preincubated with HLM for 2 min at 37 °C and the following steps of the assay was performed as outlined above.

#### Carboxylesterase incubations

The protocol for the metabolism of AMB-FUBINACA by recombinant CES1 was derived from Shimizu et al. [33] and performed as above; however, no NADPH was incorporated and recombinant CES1 or CES2 (0.1 mg/mL) was used instead of HLM. AMB-FUBINACA metabolism was quenched at 30, 60, 120 and 300 s by addition of 150 μL of ice-cold methanol. Either digitonin (100 μM; concentrations derived from Shimizu et al. [33]) or Δ^9^-THC (10 μM) was added to subsequent 120 s incubations prior to preincubation to determine inhibition of AMB-FUBINACA metabolism. Δ^9^-THC has previously been shown to inhibit oseltamivir phosphate hydrolysis by CES1 (*K*_i_ = 0.541 ± 0.081 μM) [34]; concentrations of Δ^9^-THC were selected to be significantly greater than the reported *K*_i_ values to firstly determine if an inhibitory effect was present. For all in vitro metabolism experiments, the reaction tubes were centrifuged at 14,000 g for 10 min. The following controls were also used: blank controls containing no AMB-FUBINACA, negative controls contained no microsomes or recombinant enzymes and vehicle controls that contained the corresponding solvent(s).

Experiments determining the ability of digitonin and telmisartan to inhibit CES1 and CES2 hydrolysis of PNPA were derived from Shimizu et al. [33]. CES1 or CES2 inhibition was screened by preparing phosphate buffer (100 mM), BSA (0.2% w/v), CES-1/2 (0.1 mg/mL) and either digitonin (0–100 μM) or telmisartan (0–50 μM). The reaction vessel was kept on ice at all times prior to mixing and preincubation (2 min at 37 °C). The reaction was initiated by the addition of pre-warmed PNPA (250 μM) and the total reaction volume was 200 μL. After 5 min incubation, the reaction was terminated by addition of ice-cold methanol (100 μL). Catalytic activity was quantified via *p*-nitrophenol formation by absorbance at 405 nm.

#### CB_1_ radioligand binding

Radioligand binding was carried out as previously described [2]. Briefly HEK cells expressing human CB_1_ receptors *N*-terminally tagged with preprolactin signal sequence (pplss) and 3HA (3 × haemagglutinin) epitopes were harvested in 5 mM EDTA in phosphate buffered saline (PBS), and “P2” membranes were prepared in sucrose buffer. [^3^H]-CP55,490 (1 nM) non-radiolabelled drugs, and P2 membrane preparations (3 μg/point) were diluted in binding buffer (50 mM HEPES at pH 7.4, 1 mM MgCl_2_, 1 mM CaCl_2_, 2 mg/mL BSA) and dispensed into 96‐well and V‐well plates in a final reaction volume of 200 µL (membranes were dispensed last). The plate was incubated for 1 h at 30 °C. During the incubation, a 96 well Harvest plate was treated with 0.1% w/v branched polyethyleneimine in water. Immediately prior to washing, polyethyleneimine was washed through the filters using a vacuum manifold and all wells were rinsed once with ice-cold wash buffer (50 mM HEPES at pH 7.4, 500 mM NaCl, 1 mg/mL BSA). Equilibrated binding mixture was then transferred to the Harvest plate under vacuum. Binding wells were rinsed once with wash buffer and wash solution transferred to the Harvest plate, and then wells were all washed three more times with 200 µL of wash buffer. The plate was then removed, and filters allowed to dry overnight. The next day, the plate bottom was sealed, and 50 µL of Ultima Gold XR scintillation fluid (PerkinElmer) was dispensed to each well. The plate top was then sealed, and the plate was loaded into a 96 well “rigid” cassette and loaded into a MicroBeta2^®^ TriLux Liquid Scintillation Counter (PerkinElmer). Scintillation was detected after a 30 min delay, for 2 min per well. Counts were corrected for detector efficiency.

#### CB_1_ activity via cAMP

Assays for inhibition of cAMP were performed as previously described in Finlay et al*.* [2] and Cawston et al. [35]. In brief, HEK293 cells stably expressing 3HA-tagged human CB_1_ were seeded in 10 cm cell culture dishes in normal growth medium (high glucose Dulbecco’s Modified Eagle Medium (DMEM) supplemented with 10% foetal bovine serum) and grown overnight at 37 °C in a humidified incubator (5% CO_2_). Cells that were ~ 50% confluent were transfected with the BRET CAMYEL biosensor [36]. Specifically, 30 µg of linear polyethyleneimine (dissolved in MilliQ and filter-sterilised) was diluted in 150 mM sterile saline, and 5 µg DNA was added (for a total volume of 500 µL per transfection). The transfection mixture was incubated at room temperature for 10 min, then dispensed onto the cells, mixed gently, and then returned to the incubator overnight. The next day, cells were lifted from the plate with trypsin, and plated in normal growth medium in 96-well CulturPlates (PerkinElmer) which had been pre-coated with poly-D-lysine (0.05 mg/mL in PBS for 30 min, then aspirated and washed with PBS prior to plating), then cultured overnight. Culture medium was then aspirated, and wells were washed once with warm PBS. The wash was then aspirated and replaced with 70 µL per well of assay medium (high glucose phenol-free DMEM with 25 mM HEPES, supplemented with 1 mg/mL BSA). The assay plate was returned to the incubator for 30 min, then 10 µL of freshly diluted coelenterazine-H was dispensed in assay medium (final concentration of 5 µM), and the plate was incubated in a pre-warmed plate reader (BMG Labtech, Ortenberg, Germany) for 5 min. Drugs were prepared in advance at tenfold concentration in assay medium; forskolin (or vehicle, DMSO) was combined in equal volumes with AMB-FUBINACA or AMB-FUBINACA acid (vehicle-controlled) in V-well dispensing plates. At the end of the coelenterazine-H preincubation, 20 µL of the combined drugs were dispensed (for a final stimulation volume of 100 µL per well) and the plate was read for approximately 20 min in the plate reader with simultaneous BRET1 filters (475–30 and 353–30). Data were exported into the GraphPad Prism (GraphPad Software, San Diego, CA, USA) and kinetic data were transformed into concentration–response curves using area-under-the-curve analysis.

### Data analysis

GraphPad Prism software 9.1.0 was used for statistical analysis of results. Half-life values of AMB-FUBINACA were determined using non-linear fit dissociation-one phase exponential decay. An ordinary one-way analysis of variance (ANOVA) followed by Tukey’s multiple comparisons was used to determine significance between three or more treatment groups. Statistical significance was defined as *p* < 0.05. Data are expressed as the mean ± SEM relative to negative (no metabolism) control and total inhibition percentage (inhibitor candidates only). Replicates were performed for all experiments. Experiments assessing the effect of inhibitors on AMB-FUBINACA metabolism with HLM or recombinant CES1 were replicated independently at least three times; all other experiments included some form of technical replication.

## Results

### Metabolism and inhibition in HLM

**Fig. 2:**
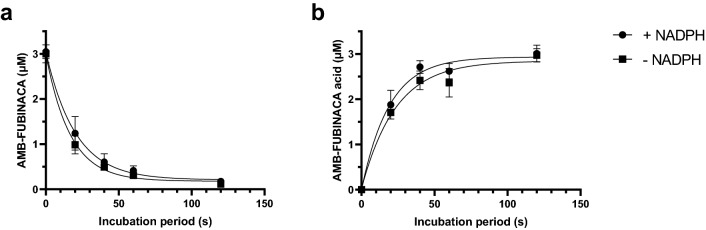
Time course of AMB-FUBINACA metabolism and AMB-FUBINACA acid formation with human liver microsomes (HLM). HLM were incubated with AMB-FUBINACA (3 μM) in the presence and absence of NADPH and **a** AMB-FUBINACA metabolism and **b** AMB-FUBINACA acid formation were determined. Drug concentrations were determined using high performance liquid chromatography–diode array detection (HPLC–DAD) analysis at 299 nm. Data are expressed as the mean ± standard deviation (SD) of three independent experiments conducted in duplicate

An AMB-FUBINACA metabolism time course was established both in the presence and absence of NADPH to determine if this biotransformation pathway was largely CYP450 or non-CYP450 mediated. CYP metabolism is dependent on NADPH for electron transfer [37]. Therefore, if AMB-FUBINACA biotransformation was dependent on NADPH, this would indicate the involvement of CYPs. Using a previously validated HPLC method (data not shown), the metabolism of AMB-FUBINACA was similar both in the presence (*t*_1/2_ = 14.0 s, 95% CI [10.5–18.5]) and absence (*t*_1/2_ = 11.6 s, 95% CI [9.28–14.3] of NADPH (Fig. 2a). Similarly, the formation of AMB-FUBINACA acid was approximately stoichiometrically equivalent to the metabolism of AMB-FUBINACA (Fig. 2b). The lack of NADPH sensitivity removed the possibility that CYPs were the primary enzymes responsible for the metabolism of AMB-FUBINACA. Therefore, the role of the hepatic esterases CES1 and CES2 in this biotransformation process was investigated.

**Fig. 3:**
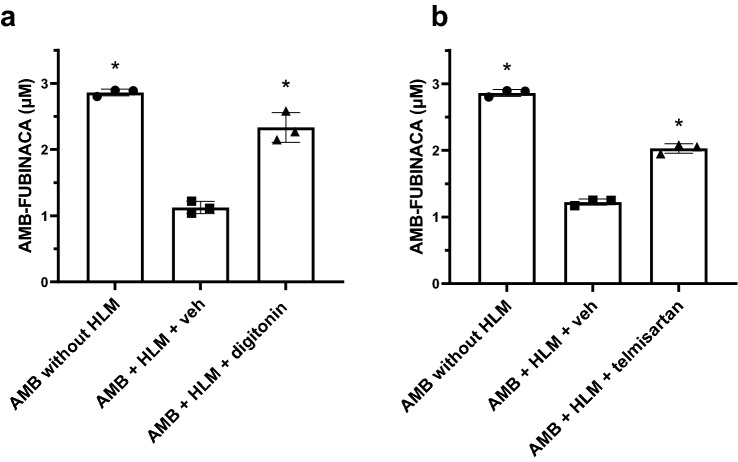
Effect of carboxylesterase inhibitors towards AMB-FUBINACA metabolism with HLM. HLM were incubated with AMB-FUBINACA (AMB; 3 μM) and either **a** digitonin **(**150 μM**)** or **b** telmisartan (50 μM**)**. Concentrations of AMB-FUBINACA were determined using HPLC–DAD analysis at 299 nm. Negative controls contained no HLM (left bars), and vehicle controls (veh; middle bars) contained the corresponding solvents. Data are expressed as the mean ± SD of three independent experiments, each conducted in duplicate. Significance was determined by a one-way ANOVA followed by Tukey’s post-hoc test; *significantly increased from the vehicle control, *P* < 0.05

CES1 and CES2 were considered, because compounds containing a methyl 3-methylbutanoate (AMB) head consistently produced an ester hydrolysis product (i.e. the corresponding carboxylic acid) as a metabolite, likely mediated by CESs [38]. To determine if CES1 or CES2 was the main metabolising enzyme of AMB-FUBINACA, an assay was performed based on the reported inhibitory specificity of digitonin and telmisartan towards CES1 and CES2, respectively [33]. Inhibition of AMB-FUBINACA metabolism in the presence of one of these inhibitors would suggest which carboxylesterase likely predominates this process. Therefore, AMB-FUBINACA metabolism was assessed over a 20 s time period in the presence of these inhibitors. The results indicated that AMB-FUBINACA metabolism was significantly reduced in the presence of digitonin (150 μM) as compared to the vehicle control (18.5 ± 3.4 vs. 60.7 ± 1.5% of total AMB-FUBINACA metabolised, respectively, *P* < 0.001, Fig. 3a). In a similar manner, telmisartan reduced the percentage of AMB-FUBINACA metabolised (29.1 ± 1.1 vs. 57.2 ± 0.7% of total AMB-FUBINACA for telmisartan and vehicle control, respectively, *P* < 0.001, Fig. 3b). These results suggest that CES1 and CES2 are both involved in AMB-FUBINACA metabolism.

### Metabolism and inhibition by recombinant carboxylesterase

**Fig. 4:**
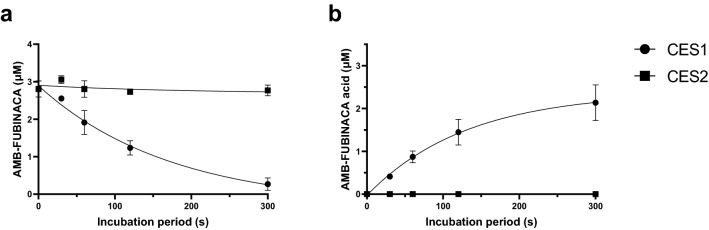
Time course of AMB-FUBINACA metabolism and AMB-FUBINACA acid formation with CES1 and CES2. Recombinant CES1 and CES2 were incubated with AMB-FUBINACA (3 μM) and **a** AMB-FUBINACA metabolism and **b** AMB-FUBINACA acid formation were determined. Drug concentrations were determined using HPLC–DAD analysis at 299 nm. Data are expressed as mean ± SD of four technical replicates

To determine the specific involvement of CES1 and CES2 in AMB-FUBINACA metabolism, time-course experiments were performed with recombinant human CES1 or CES2 (Fig. 4). The *t*½ of AMB-FUBINACA in the presence of CES1 was 120 s (*R*^2^ = 0.955). Following a 5 min incubation, only 9.5 ± 2.9% (0.27 ± 0.03 μM) of the AMB-FUBINACA remained. In contrast, no apparent metabolism of AMB-FUBINACA occurred in the presence of CES2. Specifically, following a 5 min incubation, 97.1 ± 5.8% (2.81 ± 0.11 μM) of the AMB-FUBINACA remained. Furthermore, no AMB-FUBINACA acid was detected in the presence of CES2. No metabolites other than AMB-FUBINACA acid were generated (HPLC data not shown). These results suggest that CES1 is the primary enzyme involved in AMB-FUBINACA biotransformation and that contribution by CES2 is unlikely.

**Fig. 5:**
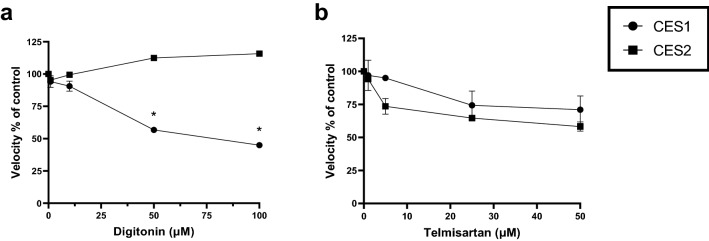
Inhibition of PNPA hydrolysis with recombinant carboxylesterases. CES1 and CES2 were incubated with PNPA (250 μM) in the presence of **a** digitonin (0–100 μM) and **b** telmisartan (0–50 μM). Control activities of CES1 and CES2 were 296 and 221 nmol/min/mg, respectively. Velocity was defined as $\frac{\mathrm{Absorbance }\left(405\mathrm{ nm}\right)}{\mathrm{Incubation period }(\mathrm{min})}$. Data are expressed as mean ± standard error of the mean (SEM) of triplicate experiments. Significance was determined by a one-way ANOVA followed by Tukey’s post-hoc test; *significantly decreased compared to CES2, *P* < 0.05

Given the discrepancy between the inhibitor studies in HLM, suggesting a role for both CES1 and CES2 in AMB-FUBINACA metabolism (Fig. 3), and the recombinant enzyme studies suggesting solely CES1 involvement (Fig. 4), we aimed to validate results reported by Shimizu et al. [33] who initially characterised the specificity of the CES inhibitors. This experiment aimed not only to validate the specificity of the inhibitors towards the respective CES isoforms, but also to confirm the activity of CES2, as no activity was observed in the prior experiment (Fig. 5). The results showed that at 100 μM of digitonin the maximal reduction in hydrolysis was observed (i.e. reaction velocity was reduced to 44.9 ± 1.9% of the control) when incubations were performed with CES1 (Fig. 5a). Importantly, digitonin did not inhibit PNPA hydrolysis by CES2 at any concentration tested. At 100 μM, the reaction velocity was 116 ± 1.6% of the control (Fig. 5a). On the other hand, telmisartan (1–50 μM) reduced metabolism of PNPA via both CES1 and CES2 (Fig. 5b). Maximal reduction in the velocity of PNPA hydrolysis in both CES1 and CES2 was observed at 50 μM (i.e. 71.0 ± 7.3 and 58.2 ± 2.5% of the control, for CES1 and CES2, respectively). At lower concentrations (5 μM), telmisartan reduced PNPA metabolism > fivefold by CES2 to a greater extent than CES1.

**Fig. 6:**
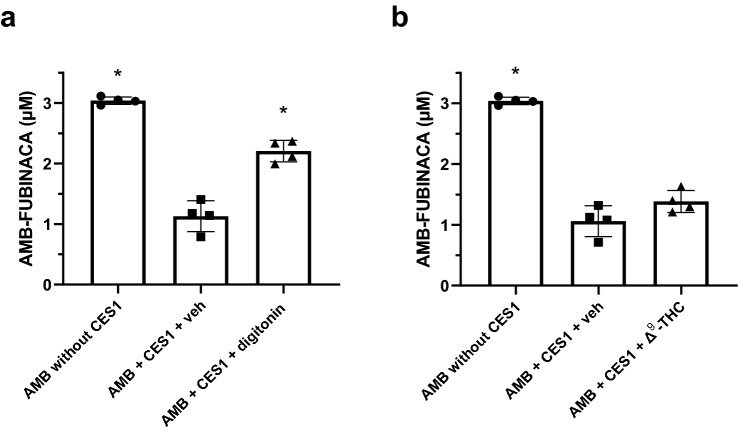
Effect of digitonin and Δ^9^-THC on AMB-FUBINACA metabolism with CES1. Recombinant CES1 was incubated with AMB-FUBINACA (AMB; 3 μM) in the presence of **a** digitonin (100 μM**)** or **b** Δ^9^-THC (10 μM**)** for 2 min. Concentrations of AMB-FUBINACA were determined using HPLC–DAD analysis at 299 nm. Negative controls contained no HLM (left bars), and vehicle controls (veh; middle bars) contained the corresponding solvents. Data are expressed as the mean ± SD of four independent experiments, each conducted in duplicate. Significance was determined by a one-way ANOVA followed by Tukey’s post-hoc test; significatnly increased from the vehicle control, *P* < 0.05

To confirm that inhibition of CES1 would impact AMB-FUBINACA metabolism, digitonin was included in the functional 120 s incubations used initially. Δ^9^-THC was also used as a biologically relevant control, given that it has been previously reported to be a CES1 inhibitor [34] and it was detected in the blood of 41% of the 58 fatal cases in New Zealand. The results showed that digitonin significantly reduced total percentage of AMB-FUBINACA metabolised by CES1 as compared to the vehicle control (27.5 ± 2.9% vs. 62.8 ± 4.2% of total AMB-FUBINACA metabolised, respectively, *P* < 0.001; Fig. 6a). Interestingly, there was no statistically significant difference in AMB-FUBINACA metabolism by CES1 between vehicle control and Δ^9^-THC (Fig. 6b). However, Δ^9^-THC inhibited AMB-FUBINACA metabolism only by ~ 16%.

### CB_1_ binding and activity

**Fig. 7:**
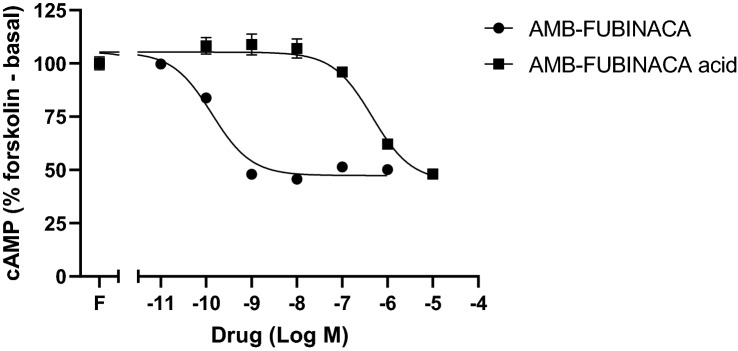
Concentration response-curves for AMB-FUBINACA and AMB-FUBINACA acid cAMP signalling at CB_1_ receptor. HEK cells stably expressing 3HA-hCB_1_ were incubated with AMB-FUBINACA (10 pM–1 µM) and AMB-FUBINACA acid (0.1–10 µM) in the presence of 5 µM forskolin. The BRET assay was conducted for 20 min in a plate reader with simultaneous BRET1 filters (475–30 and 535–30 nm). Data were then exported into GraphPad Prism software and kinetic data were transformed into concentration–response curves using area-under-the-curve analysis. Values are the mean ± SEM from a representative experiment performed in duplicate

AMB-FUBINACA has previously been reported to be a high affinity agonist of CB_1_ with a *K*_d_ of 1.9 nM [2]. In contrast, very low binding affinity was observed for AMB-FUBINCA acid, such that 10 µM only displaced 25.7 ± 7.3% (*n* = 3) of the radioligand. To compare CB_1_ receptor-mediated activity of AMB-FUBINACA and its major metabolite AMB-FUBINACA acid, cAMP signalling was characterised. As CB_1_ receptor signalling is canonically mediated through Gα_i/o_ proteins, agonism results in adenylate cyclase inhibition, subsequently reducing cAMP levels. As expected, AMB-FUBINACA inhibited forskolin-induced cAMP with sub-nanomolar potency (pEC_50_ 9.83 ± SEM 0.04, *n* = 3) (Fig. 7). AMB-FUBINACA acid also showed agonism but with > 3000-fold lower potency (pEC_50_ 6.35 ± SEM 0.03, *n* = 3). However, both compounds produced similar maximal effects with *E*_MAX_ values of 57.0 ± 1.6% and 58.6 ± 1.7% (both *n* = 3), for AMB-FUBINACA and AMB-FUBINACA acid, respectively.

## Discussion

The metabolism of AMB-FUBINACA and subsequent formation of AMB-FUBINACA acid occurred largely in the absence of the cofactor NADPH, suggesting this biotransformation process was not CYP450-mediated. While this study and other work [24] demonstrate the rapid metabolism of AMB-FUBINACA and formation of the demethylated metabolite AMB-FUBINACA acid, many other studies used incubation periods of 60 min or greater [14, 29, 39].

Extended incubation periods may underestimate the concentrations of AMB-FUBINACA acid due to the production of other metabolites, as depicted by the metabolic pathway proposed by Xu et al. [39]. This additional metabolism may have resulted in an underestimation of the dominance of AMB-FUBINACA acid, though it should also be noted that the acid metabolite has been observed to remain stable for at least 120 min in a HLM metabolism assay [25]. However, the fact that the experimental *t*_1/2_ of AMB-FUBINACA in HLMs in this study (14.03 s) was significantly less than that of Brandon et al. [26] (5.9 ± 0.48 min) highlights the importance of sampling at short incubation periods to characterise initial metabolism. Additionally, the relevance of subsequent metabolism of AMB-FUBINACA is unclear, as only the acid metabolite has been identified in the majority of studies in humans. Additionally, the protein concentration of HLM in this work was fourfold less than that used in similar studies [14, 25, 29, 39]. Initial experiments at a protein concentration of 1 mg/mL caused marked metabolism of AMB-FUBINACA, but concentrations were below the limit of detection after a 5 min incubation period. Reproducible and quantifiable metabolite production was achieved when the protein concentration of HLM was reduced to 0.25 mg/mL and the incubation period ranged from 20 to 120 s. Thus, it is recommended that in further work very short incubation periods and lower protein concentrations are used to accurately determine the effect of other drugs and chemicals on the metabolism of AMB-FUBINACA.

Preincubation of HLM with the proposed selective CES1 inhibitor digitonin and the selective CES2 inhibitor telmisartan [33] resulted in a significant reduction in AMB-FUBINACA metabolism. If these inhibitors were truly selective, this would have indicated that AMB-FUBINACA metabolism was mediated by both CES1 and CES2. Subsequent experiments confirmed that digitonin significantly reduced PNPA hydrolysis via CES1 to ~ 45% of the control. Telmisartan, on the other hand, did not show a high degree of specificity towards CES2, as at 50 μM, activity of CES1 was reduced to ~ 71% of control and activity of CES2 was reduced to ~ 58% of control (Fig. 5). This implies that telmisartan does inhibit CES2, but it is not selective for CES2. However, a more complete picture could be obtained by performing a complete range of concentration–response experiments. Importantly, metabolism of AMB-FUBINACA by recombinant CES1A1 and CES2 confirmed the involvement of CES1. Metabolism occurred significantly faster by HLM (*t*_1/2_ = 11.6 s) as compared to CES1 (*t*_1/2_ = 120 s) which may be related to the differences in total enzyme content in the experimental preparation. Liver microsomes contain various isoforms of both hepatic CYP450 and non-CYP450 enzymes. However, recombinant enzymes only contain a specific isoform of an enzyme [40]. While the metabolism of AMB-FUBINACA has been confirmed with CES1A1, additional CES1 isoforms present within HLM may also contribute to this biotransformation process. However, the identification of CES1 as one of primary enzymes in AMB-FUBINACA metabolism is an important finding as it enables the prediction of potential drug–drug interactions and may give insight into the toxicity elicited by AMB-FUBINACA in individuals who are also taking prescription and/or recreational drugs.

AMB-FUBINACA metabolism by recombinant CES1A1 was inhibited by ~ 56% when incubated with 100 μM digitonin. However, Δ^9^-THC (10 μM) did not significantly affect metabolism despite concentrations nearly 20-fold greater than the reported *K*_i_ value being tested [34]. Previously the effect of Δ^9^-THC upon CES1 was examined in human liver S9 fractions, measuring oseltamivir phosphate hydrolysis [34]. S9 fractions contain both microsomal and cytosolic enzymes and thus CES1 concentrations in S9 fractions are significantly lower than recombinant preparations [41]. Since the final protein concentrations in this study exceed those used by Qian et al. [34] by fivefold (20 vs. 100 μg/mL), it is possible that there is residual metabolic activity associated with the higher protein. However, *K*_i_ is a measurement of affinity that is independent of protein concentration [42]. Thus, it is also possible that CES1 has multiple catalytic sites which are substrate-specific. Consistent with this idea, CES1 has been reported to contain three ligand-binding sites, an active site, a “side door” and a Z-site [43]. However, there are no obvious explanations as to why Δ^9^-THC inhibited CES1 in studies conducted by Qian et al. [34] but failed to significantly inhibit CES1 when incubated with AMB-FUBINACA.

AMB-FUBINACA acid displayed only moderate displacement of [^3^H]-CP55,940 at 10 µM, consistent with a p*K*_i_ > 5, or approximately 500–1000 fold less than reported for AMB-FUBINACA (p*K*_i_ = 8.72) [2]. Consistent with this, AMB-FUBINACA acid was substantially less potent at CB_1_ as compared to the parent compound, indicating the importance of an uncharged head group in promoting a ligand-receptor conformation with high efficacy [44]. Importantly, the effect of AMB-FUBINACA on cAMP was in line with previously reported results using the same cell line [2] and thus showing a consistent response. A less efficacious metabolite suggests that inhibition of AMB-FUBINACA metabolism in vivo is likely to result in prolonged CB_1_ activation by preventing metabolism of the more potent parent compound. Prolonged activation of CB_1_ has previously been linked to receptor desensitisation, internalisation, degradation, and neuronal cell death [45–47] and these effects may also contribute to the toxicity of AMB-FUBINACA.

The rapid metabolism of AMB-FUBINACA by HLM and recombinant CES1 would indicate that factors that reduce this detoxification would enhance CB_1_ pharmacological action and toxicity [48]. Other factors may include sex differences in enzyme expression, as females express ~ 17% higher levels of hepatic CES1 as compared to males [49] and genetic differences resulting in CES1 polymorphisms [50]. Interestingly, this appears to align with the higher prevalence of SCRA toxicity experienced by males. Specifically, 88% of the deaths in New Zealand attributed to AMB-FUBINACA were male [1]. Thus, it is possible that lower CES1 expression may result in a prolonged exposure to AMB-FUBINACA and thus greater toxic events. However, males also use new psychoactive substances and SCRAs such as AMB-FUBINACA at a higher frequency than females [51, 52]. Further investigations should probe the potential sex-dependent differences in AMB-FUNBINACA metabolism.

## Conclusions

AMB-FUBINACA metabolism resulted in the rapid formation of AMB-FUBINACA acid; this did not require the CYP450 cofactor, NADPH. CES1A1 was identified as one of the primary metabolising enzymes of AMB-FUBINACA, whereas CES2 failed to metabolise it. Furthermore, metabolism of AMB-FUBINACA by HLM was inhibited by digitonin and telmisartan to varying degrees, likely via inhibition of CES1. Although Δ^9^-THC had been reported as a CES1 inhibitor, this study found that it did not modulate the metabolism of AMB-FUBINACA by CES1. The potency shift of AMB-FUBINACA acid relative to the parent compound at CB_1_ would suggest that physiological effects produced by the acid metabolite would be minimal, and toxicity is likely mediated by AMB-FUBINACA. Inhibition of AMB-FUBINACA metabolism would increase CB_1_- and non-CB_1_-mediated effects in humans by preventing the production of a less potent metabolite.
